# Attenuated but immunostimulatory *Mycobacterium tuberculosis* variant *bovis* strain Ravenel shows variation in T cell epitopes

**DOI:** 10.1038/s41598-023-39578-5

**Published:** 2023-07-31

**Authors:** Evan P. Brenner, Srinand Sreevatsan

**Affiliations:** Department of Pathobiology and Diagnostic Investigation, College of Veterinary Medicine, Michigan State University, 784 Wilson Road, East Lansing, MI 48824 USA

**Keywords:** Computational biology and bioinformatics, Immunology, Microbiology, Molecular biology, Molecular medicine

## Abstract

Tuberculosis, caused by *Mycobacterium tuberculosis* complex (MTBC) organisms, affects a range of humans and animals globally. Mycobacterial pathogenesis involves manipulation of the host immune system, partially through antigen presentation. Epitope sequences across the MTBC are evolutionarily hyperconserved, suggesting their recognition is advantageous for the bacterium. *Mycobacterium tuberculosis* var. *bovis* (MBO) strain Ravenel is an isolate known to provoke a robust immune response in cattle, but typically fails to produce lesions and persist. Unlike attenuated MBO BCG strains that lack the critical RD1 genomic region, Ravenel is classic-type MBO structurally, suggesting genetic variation is responsible for defective pathogenesis. This work explores variation in epitope sequences in MBO Ravenel by whole genome sequencing, and contrasts such variation against a fully virulent clinical isolate, MBO strain 10-7428. Validated MTBC epitopes (n = 4818) from the Immune Epitope Database were compared to their sequences in MBO Ravenel and MBO 10-7428. Ravenel yielded 3 modified T cell epitopes, in genes *rpfB*, *argC*, and *rpoA*. These modifications were predicted to have little effect on protein stability*.* In contrast, T cells epitopes in 10-7428 were all WT. Considering T cell epitope hyperconservation across MTBC variants, these altered MBO Ravenel epitopes support their potential contribution to overall strain attenuation. The affected genes may provide clues on basic pathogenesis, and if so, be feasible targets for reverse vaccinology.

## Introduction

Over 100 years since vaccination by *M. tuberculosis* variant *bovis* (MBO) strain BCG began, tuberculosis remains the deadliest single infectious agent in the world for humans. Our understanding of *Mycobacterium tuberculosis* complex (MTBC) pathogenesis remains lacking, both in humans and in the myriad non-human hosts MTBC variants attack. An underlying mechanism behind this is the subversion and misdirection of the host immune response, achieved both directly by targeted alteration of host kinase signaling cascades, and indirectly by intentional presentation of conserved antigens that drive specific immune feedback^1–4^. In a background of mycobacterial antigenic hyperconservation, this research asked what level of epitope variation can be detected in each an attenuated and a virulent MBO strain, and if variation may signal loss of virulence through dysregulation of host immune control.

At a simplistic level, a host’s immune response to infection—including recognition of pathogen markers, response by innate and adaptive immunity, and ultimate pathogen clearance—depends on the receipt of signals differentiating self and foreign molecules. Antigens here generically refer to such markers that a host can react to in mounting a pathogen-specific immune response. Under the classic model, after initial infection, pathogen antigens recognized by the immune system are under selective pressure to change, and the host in return faces selective pressure to maintain recognition of changing targets in a process long referred to as an evolutionary arms race^5–7^. Over the course of the infection, immunity develops, targeting of specific antigens arises, the infection is suppressed, and re-infection by the same pathogen later hindered. On the other hand, immune responses to mycobacterial pathogens like *M. tuberculosis* are well-known to be more nuanced and skewed towards a cell-mediated immune response^4,8,9^. Early research suggested a limited role of B cells or antibodies in protection, and although these subsets do have beneficial roles^3,10,11^, it is known that a successful immune response against MTB infection absolutely requires CD4^+^ and CD8^+^ T cell activity^2,3,12^. Subsequent work has focused heavily on a process of initial engulfment of MTB by macrophages, subsequent intracellular MTB replication and cell signaling that leads to a Type IV hypersensitivity response wherein T cells contribute towards granuloma formation to seal in the antigen provoking the response^2–4^. In most infections, this successful response drives the granulomatous encasement of MTB, not its sterilization^8,13^. While most patients with latent TB infection (LTBI) do not progress again to active disease, MTB is able to survive indefinitely within the confines of the granuloma, and in reactivation, MTB drives caseation of the granuloma core and subsequent spillage of bacilli from the granuloma into the lung, allowing dissemination and respiratory transmission to others^2,3,13,14^. This reactivation is again believed to involve MTB host manipulation by antigen presentation in a hypersensitized host state, and is not associated with an increased bacterial load, indicating some degree of bacterial control over the host immune response by antigen presentation drives disease^13,14^. Amidst this backdrop, it has been observed by multiple groups that T cell epitopes in *M. tuberculosis* are hyperconserved, equivalent to that seen in the most essential MTB genes^6,15^. This supports a model wherein T cell recognition of MTB epitopes is essential to bacterial survival^4,6,13,15^. After millennia of coevolution in human hosts, MTB has developed a strategy of immune subversion not rooted in antigenic obfuscation, but in the intentional presentation of conserved targets that manipulate the host immune system. This triggers unproductive immune responses that, at least at a population-scale, confer fitness benefits for the pathogen^13,16^. In short, T cell responses are necessary to control tuberculosis, but MTB has also evolved to exploit these same responses, provoking specific immune responses that can ultimately benefit MTB and perpetuate disease^6,10,13,15,17^.

Given the specialization of pathogenic mycobacteria into manipulation of the host by intentional presentation of specific, hyperconserved T cell epitopes, this work posits that evidence of attenuation and adaptation can be found in epitope variation. Changes observed in known T cell epitopes in attenuated strains may represent a contributor to such attenuation, and identification of such epitopes would therefore be informative for pathways necessary for mycobacterial subversion of host immune responses. While most vaccine strategies continue to target immunodominant antigens like ESAT-6 or the Antigen 85 complex, the identification of other antigens where variation is associated with dysregulation of host immune manipulation could lead to selection of better, more protective targets.

**Figure 1:**
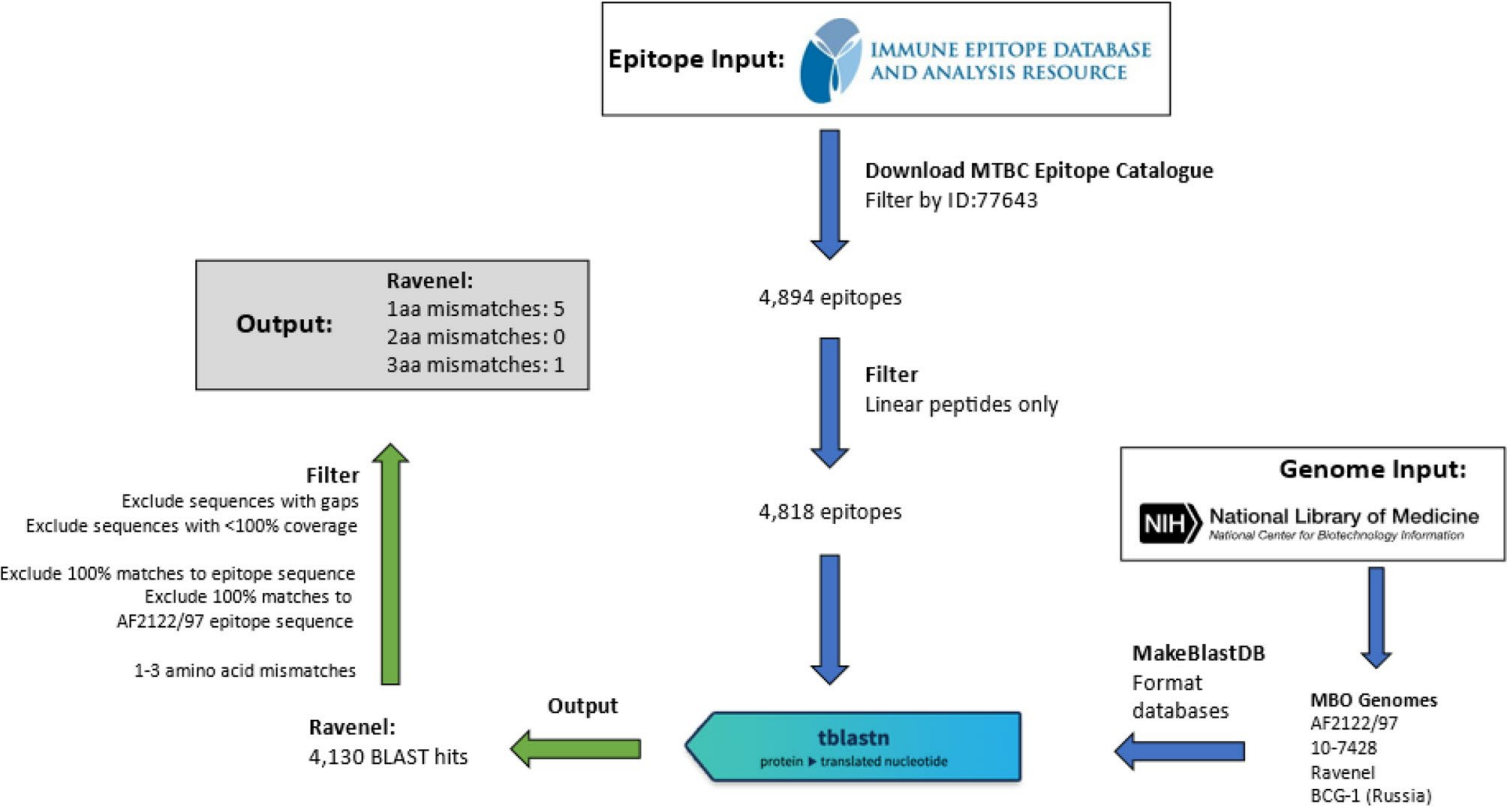
Schematic of epitope extraction workflow for MBO strain Ravenel. Epitope Input: All classified epitope sequences from any *Mycobacterium tuberculosis* complex members are downloaded and filtered to include linear peptide sequences. Genome Input: The MBO reference genome AF2122/97, as well as reference BCG-1 (Russia) genomes were downloaded in .fna format from NCBI RefSeq. Strains Ravenel and 10-7428 were downloaded as draft genomes. Genomes were processed by BLAST + command makeblastdb to generate searchable BLAST databases. tblastn: Blast searches were performed with default parameters, searching 4818 epitopes against each genome database to match IEDB epitopes to their counterparts encoded in the genome. Output and Filtering: Blast hits were filtered as described in “Materials and Methods” to identify epitopes with amino acid substitutions vs. the IEDB epitope and that are not seen in reference AF2122/97.

This work sought to analyze variation of known MTBC epitopes between a subset of virulent and attenuated *M. tuberculosis* variant *bovis* (MBO) strains, with a hypothesis that variation in T cell epitopes will be more frequent in attenuated strains, and that these variant epitopes may contribute to observed attenuation. To explore this topic, polymorphisms were extracted from the recently sequenced genomes of MBO strain Ravenel, a naturally attenuated cattle strain that does not carry the causative genomic lesions—large, contiguous deletions of genetic material like RD-1—of BCG strains, and MBO strain 10-7248, a fully virulent cattle clinical isolate^18–20^. For additional comparison, the MBO strain BCG-1 (Russia) vaccine strain believed closest to the now-lost ancestral BCG strain^21,22^ and the MBO strain AF2122/97 reference ^23,24^ were analyzed, the former to assess epitope variation in a truly dysfunctional MBO strain, and the latter to exclude variation fixed in the MBO genetic background for any epitopes characterized only in non-MBO complex members. Validated MTBC epitopes collected from the Immune Epitope Database (IEDB) were compared against SNPs in the genomes of the four selected strains, with a focus on 1–3 amino acid changes to represent variation through point mutations, and to limit both nonspecific hits on other epitopes and the analysis of more extensive variations known to exist in PE/PPE genes contrary to the overall pattern of epitope conservation^7^. The analysis is represented by the schematic in Fig. 1.

## Results

For Ravenel, 4130 raw epitope hits were recorded against the IEDB dataset (Table 1: Raw Hits). Of these, 2488 were a perfect match to a characterized epitope, which are considered uninformative as this work seeks epitope variation (Table 1: Matches). In Ravenel, filtering the remaining subset to epitopes showing a single amino acid mismatch vs. a characterized epitope, 100% coverage of the Ravenel hit vs. the IEDB query, and with no gaps vs. the IEDB query returned 303 matches (Table 1: Degenerate Mismatches). In most cases in the epitope dataset, sequences are *M. tuberculosis* var. *tuberculosis*-derived, and additionally, one epitope can have multiple known variants in the database—thus, degenerate mismatches—and so each mismatch was then interrogated for a perfect match against other variant epitope sequences, as well as against the AF2122/97 epitope hits to reduce the chance variants were simply fixed in the MBO background. Of the 303 Ravenel single amino acid hits against the epitope dataset, 188 mismatches were found to have a 100% match against a different variant epitope in the dataset and were therefore excluded, leaving 115 epitopes in Ravenel that differed by 1 amino acid from the IEDB sequences. Comparing these 115 epitopes to the output for the epitope workflow for AF2122/97 yielded 100% matches for 110, leaving just 5 epitopes in Ravenel that were mismatches from known sequences, and different from the same epitope sequence in the MBO reference strain AF2122/97 (Table 1: Unique Mismatches). Expanding to 2 amino acid mismatches yielded 293 degenerate epitopes, and 0 unique epitopes. Finally, 3 mismatches returned 289 degenerate epitopes, and filtered to 1 unique epitope. This process was repeated for 10-7428 and BCG-1. It is important to note that for reference AF2122/97, the process does not include subtracting epitopes that may be the wild-type in MBO, yielding an artificially high count of 121 1AA changes, 28 2AA changes, and 13 3AA changes. Table 1 reports the initial results for each of the 4 strains.

**Table 1:**
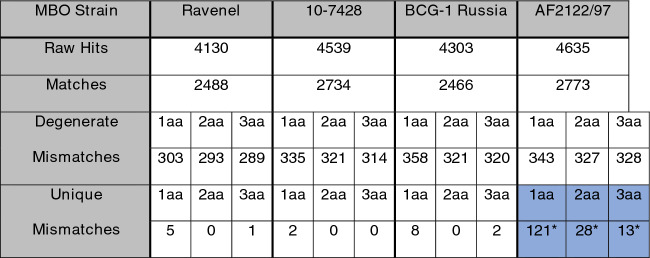
Epitope homology results for four genomes. *M. tuberculosis* var. *bovis* strains AF2122/97 (virulent, reference), BCG-1 Russia (attenuated, reference), 10-7428 (virulent, newly sequenced), and Ravenel (attenuated, newly sequenced). Epitopes (n = 4894) were selected from IEDB.org and filtered to 4818 to query against the 4 genomic .fna files indexed for tblastn. The raw hits row indicates the number of epitopes aligning anywhere in the genome designated per column. Matches indicates 100% similarity to at least one epitope variant in the IEDB dataset. Mismatches indicates alignment but with #aa differences (indicated per column, 1, 2, or 3 mismatches). Finally, Unique Mismatches indicates the mismatched sequence does not also map perfectly to any other variant epitope sequences from IEDB or, with the exception of blue-shaded AF2122/97 cells, that these changes are not observed in AF2122/97 epitopes either. Sequences with gaps or with more than 3 mismatches are not included in the analysis, so values do not sum to the original Raw Hits value. *AF2122/97 values are not subtracted from what may be MBO wild-type variation like other strains are, and thus its values should not be compared directly.

Most mismatches in Ravenel, 10-7428, and BCG-1 mapped perfectly to an existing variation in AF2122/97, leaving only a handful of changes not observed elsewhere (Table 1: Unique Mismatches). In Ravenel, five potentially impactful single amino acid mismatches were initially recorded. Epitope ID 229352, a 15aa epitope and one of only two known antigenic regions in RpfB, showed Glu263Gly. Epitope 595988 in ArgC presented Tyr20His. Epitope 597585 in RpoA showed Glu75Asp. These substitutions are provided as Supplemental File 1: Ravenel Changes. The remaining two mismatches (in epitopes 163642 and 163423) both affected the same gene, *esxJ*, but this region showed an unusual pattern in the Ravenel assembly with the gene broken into 3 ORFs each containing partial starts and stops, with an identical broken pattern seen in the 10-7428 assembly suggesting this may be a systemic assembly error, and so changes in this gene were marked as unreliable. The three amino acid mismatch in Ravenel (epitope 100593) was also unreliable, mapping to an unknown PE/PPE family gene on an unplaced contig. These excluded sequences are listed in Supplemental File 1: Ravenel Unreliable Changes. In contrast, virulent strain 10-7428 showed a single 1 amino acid substitution, PPE42 Asn232Asp in B cell epitope 10022 (Supplemental File 1: 10-7428 Changes). Strain 10-7428 showed a 3 amino acid mismatch beyond this, again in partial *esxJ* hits could be an assembly error (Supplemental File 1: 10-7428 Unreliable Changes) which led to subsequent exclusion of this epitope.

As expected, BCG-1 Russia showed the most variation in this analysis, a finding that likely arises both from its historical age^21,22^ relative to the others in the dataset as well as its impaired functionality. Antigen 85B showed a substitution Phe140Leu that impacted four overlapping epitopes; PPE genes featured multiple variants, including three epitopes inconclusively from the highly homologous PPE18/PPE19 cluster of genes known to show sequence variation compared to their characterized T-cell epitopes^7^ and one in PPE25; the virulence-associated serine protease MarP showed one; and one change was observed in an Mce family protein (Supplemental File 1: BCG-1 Changes).

The values for AF2122/97 are relative to the data from IEDB with is predominated by MTB epitopes, and so its unique mismatches values appear much greater than the other strains. A more objective comparison would be analyzing degenerate mismatches (Table 1: Degenerate Mismatches), where strains appear similar.

For observed changes in Ravenel and 10-7428, predictions of effects on protein stability by ΔΔG values were performed and recorded for observed single mutations (Table 2)^25–29^. Full length protein sequences for RpfB, ArgC, and RpoA in Ravenel, and PPE42 in 10-7428 were searched by BLASTP against the AF2122/97 sequences for potential compensatory mutations, but only the originally detected single amino acid substitution was seen across the entire protein for each of these. For RpoA, given its presence in the large multi-subunit RNA polymerase complex, additional searches were performed for Ravenel’s RpoB and RpoC sequences, but these were unchanged relative to the AF2122/97 reference.

**Table 2:**
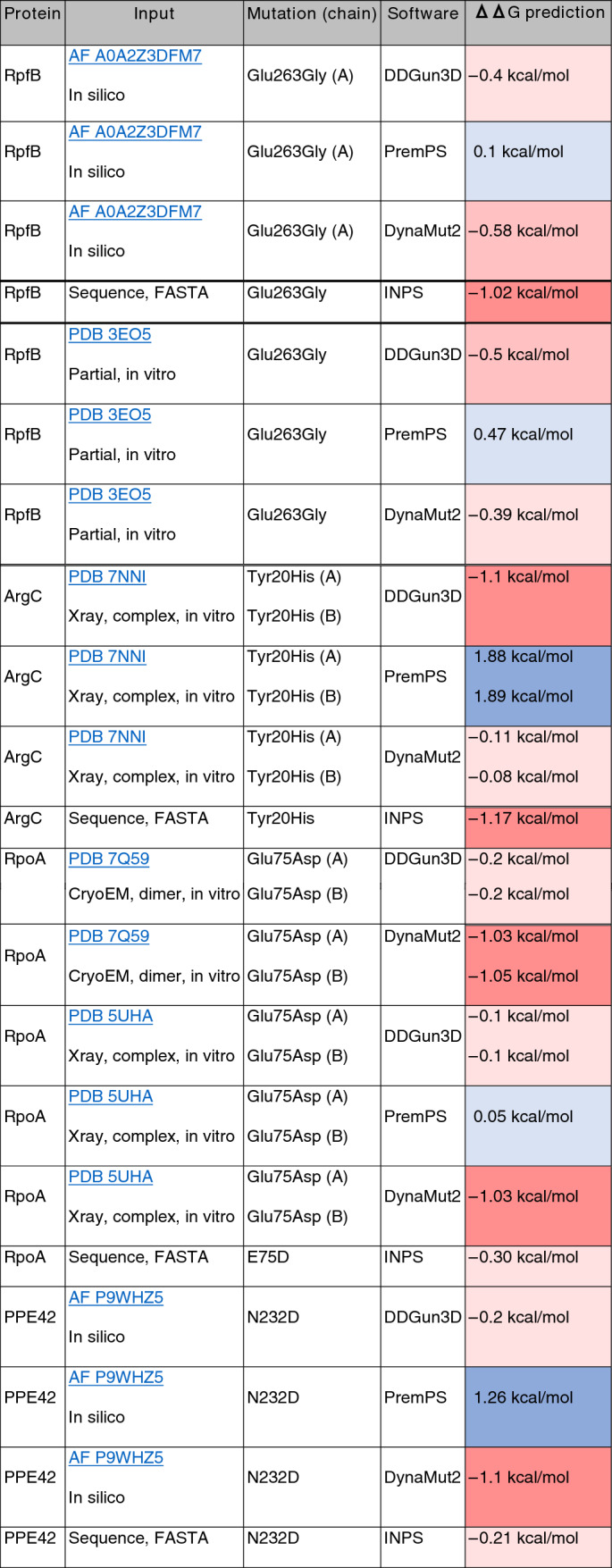
ΔΔG predictions on protein structure of a subset of identified mutations**. **Software packages DDGun3D, PremPS, and DynaMut2 utilize input of .pdb structures, though PremPS only accepts X-ray crystallography and not Cryo-EM structures. Software INPS uses sequence information exclusively. ΔΔG values are shaded from blue (+ ΔΔG) to red (− ΔΔG).

## Discussion

It is known that, with the exception of some members of the PE/PPE gene family, T cell epitopes are hyperconserved across the MTBC^6,7,15^. This is thought to reflect an evolutionary strategy of intentional host immune manipulation by presentation of T cell epitopes to drive specific immune responses that the pathogen can leverage to its advantage^6,13,15,17^. In this work, epitopes were studied to assess whether variation in T cell epitopes might be associated with attenuation by a loss of host immune modulatory potential. An initial investigation was performed using recently sequenced MBO Ravenel, an attenuated strain that provokes a robust immune response in cattle but does not cause lesions or persistent disease. In comparison, fully virulent cattle isolate MBO strain 10-7428 was also analyzed, along with MBO BCG-1 and MBO AF2122/97. Overall, the number of changes observed across the three strains compared to AF2122/97 was small, but was expected based on existing knowledge about T cell epitope conservation^6,15^. Analysis shows 3 changes to T cell epitopes across Ravenel, impacting dormancy, arginine biosynthesis, and the alpha chain of the DNA-dependent RNA polymerase. In contrast, no T cell epitope changes were seen in 10-7428, which bore only a single change in a B cell epitope of PPE42, an immunogenic gene already known to exhibit antigenic variation^7,30^. These results are compatible with the hypothesis that T cell variation could impair pathogenesis, though further investigation and confirmatory testing of these specific epitopes is required.

**Figure 2:**
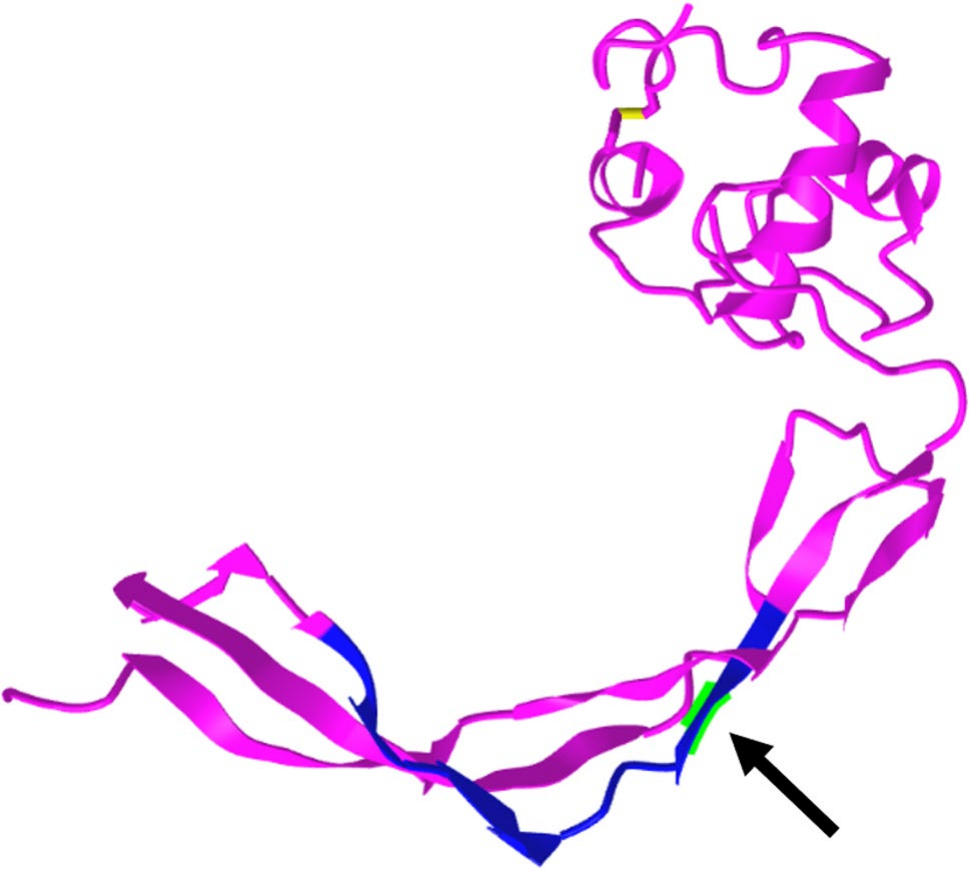
RpfB 3D structure (PDB 3EO5). Crystal structure generated by Ruggiero et al*.* (2009) by X-ray diffraction, representing RpfB residues 194–362 making up the G5 domain. Flat arrow shapes represent beta sheets, corkscrews represent alpha helices, and other parts are unstructured. Blue backbone: epitope 229352. Black arrow pointing to green highlight: Residue 263 (E263G in Ravenel).

RpfB, or resuscitation promoting factor B, is one of five *rpf* genes known to be crucial in the transition from mycobacterial dormancy back to growth and infection dissemination. It is known that deletion of any one *rpf* gene still allows normal in vitro or in vivo growth but significantly impaired reactivation in a murine model of MTB infection, and furthermore that deleterious effects in infection and persistence are dramatic in a double-knockout background^31^. MBO Ravenel is capable of cattle infection, but after provoking a strong immune response it fails to produce lesions in most experimentally infected animals, unlike virulent strains^20,32^. The change (Glu263Gly) observed in epitope 229352 was earlier reported as a SNP^20^. The large, polar, charge-bearing glutamic acid to a small, flexible, non-polar glycine is a major alteration, and across homologues, position 263 is almost always either a glutamic acid, or an aspartic acid (the COG3583 consensus sequence residue). RpfB in Ravenel is unique in NCBI’s Identical Protein Groups database, and TBLASTN searches of the NCBI NR database and WGS database filtered by *Mycobacterium tuberculosis* complex (taxid: 77643) showed no hits for this substitution outside Ravenel. Per a partial crystal structure by Ruggiero et al*.*, residue 263 (Fig. 2, green highlight) is in a linking region connecting two different three-strand stretches of beta-sheets, and the enhanced flexibility caused by replacement of this large, charged residue with a glycine could destabilize this region^33^. However, in silico ΔΔG predictions yielded inconsistent results ranging from weakly stabilizing to weakly destabilizing (Table 2: RpfB) and what effects this may have on the larger protein structure and function are uncertain. Since the catalytic residue and binding pocket is more than 30 amino acids upstream, it is unlikely that catalysis is directly compromised by this change, but the influence on tertiary structure and function is an open question that needs to be further evaluated. Regardless, this substitution is considerable at an epitope level, so even if protein functionality is maintained, altered immune recognition of the RpfB protein may still affect virulence. Epitope 229352 is T cell epitope shown previously to elicit a CD8^+^ dominant response and release of IFN-γ and TNF-α^34^. As such, RpfB remains an interesting candidate for modulation of virulence in MBO Ravenel and beyond.

**Figure 3:**
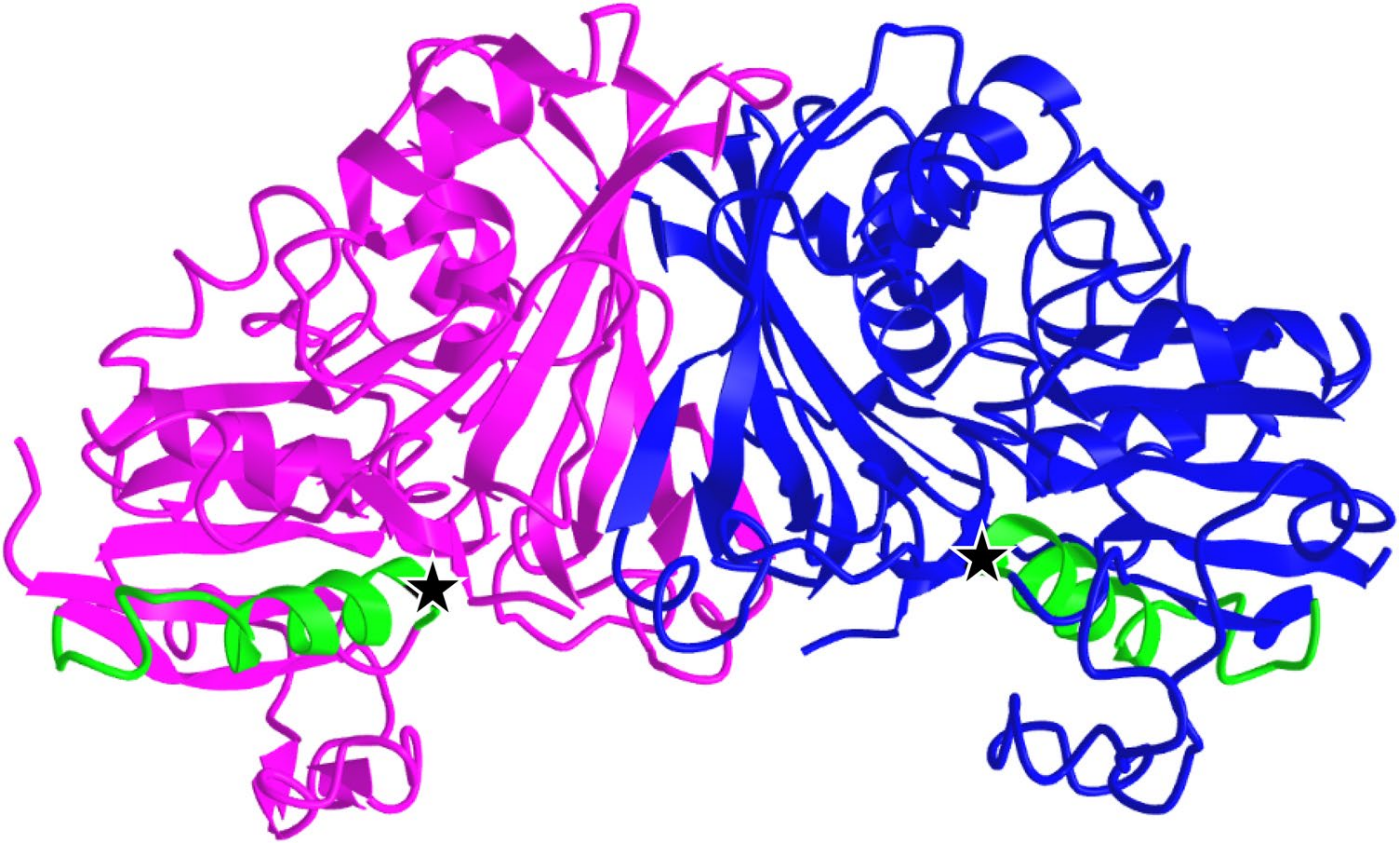
ArgC 3D structure (PDB 2NQT). Crystal structure generated by Cherney et al*.* (2006) by X-ray diffraction of full-length protein. Flat arrow shapes represent beta sheets, corkscrews represent alpha helices, and other parts are unstructured. Magenta and blue: ArgC chains A and B, respectively. Green: epitope 595988. Black stars at end of green epitopes: Residue 20 (Y20H in Ravenel). Partial complex of 2NQT shown over full complex of 7NNI used in DDG predictions for visual clarity.

ArgC, or N-acetyl-gamma-glutamyl-phosphate reductase, is an oxidoreductase found in the L-arginine biosynthesis pathway^35^ and well-conserved across Actinobacteria. Transposon mutagenesis in MTB has identified all Arg members as essential *in vitro*^36^, and *argB* and *argF* knockouts are efficiently sterilized from murine infection models in both C57BL/6 and immunocompromised SCID mice, with Δ*argB* infections of the latter group resulting in 100% survival to 300 days and complete clearance even at 10^8^ CFU/mL doses^37^. Less research has been performed with ArgC by comparison, but a modest IFN-γ response against the specific epitope 595988 has been demonstrated by ELISA in *Bos taurus*^38^. Investigation by Gupta et al*.* of MTB ArgC places Tyr20 near a structural center where it forms a hydrogen bond with Glu203^35^. Upon binding substrates, the conformation of ArgC shifts around the region of this mutation^35^, which could allow modest functional impacts despite the similar structures of tyrosine and histidine. This Tyr20His alteration (Fig. 3: black stars) in epitope 595988 (Fig. 3: green) is observed in a total of 6 MTBC isolates on NCBI: Ravenel, two human MTB isolates (2926STDY5723476, 01-R1463) a cattle MBO isolate (2008/0665), an MBO type strain (ATCC 19210), and an MBO lab isolate (strain 30). Like for RpfB, the impact of this change on a short, linear T cell epitope seems more likely significant than a tyrosine to histidine substitution for overall protein function. While this change is rare, its presence in a small number of human and bovine clinical isolates makes it unlikely to have a major impact on pathogenesis alone. It may instead be a contributor in overall attenuation through cumulative changes in T cell recognition.

**Figure 4:**
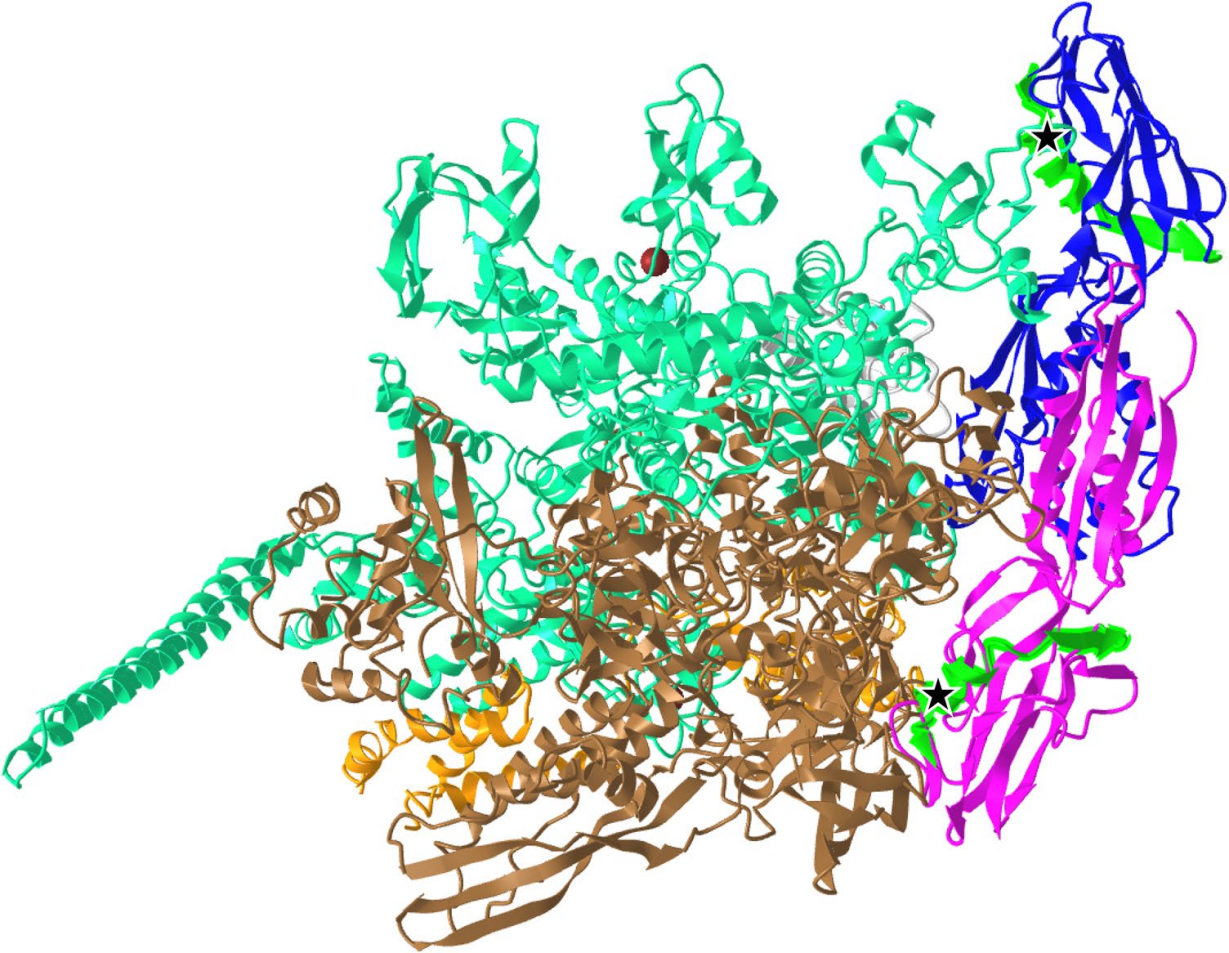
RNA polymerase complex 3D structure (PDB 5ZX3). Crystal structure generated by Li and Zhang (2019) by X-ray diffraction, representing the full RNAP complex in association with sigma factor H. Flat arrow shapes represent beta sheets, corkscrews represent alpha helices, and other parts are unstructured. Magenta and blue (right): RpoA chains A and B. Green backbone in RpoA: epitope 597585. Black stars at ends of green epitopes: residue 75 (E75D in Ravenel).

RpoA, or the DNA-directed RNA polymerase subunit alpha, is a core subunit of the RNA polymerase (RNAP) complex conserved across bacteria. It is the target of the antibiotic rifampicin, and while this critical enzyme is known to be intensely conserved, in MTB in particular, changes in RpoA-RpoC are known to be associated with rifampicin resistance, especially with changes in RpoB^39,40^. It has also been found in *Mycobacterium* as well as *Salmonella* that compensatory mutations across the genes in the RNAP complex are necessary to offset the fitness deficits of resistance-conferring mutation in these essential genes^39,41,42^. In MBO Ravenel, a change was observed in epitope 597585, leading to RpoA Glu75Asp (Fig. 4). No other substitutions are seen in RpoA, RpoB, or RpoC. Curiously, RpoA Glu75Asp is seen in all the same strains that carry ArgC Tyr20His change, as well as MBO strains M1009 and M1010, both clinical isolates from slaughter of two cattle (*Bos taurus*) in Paraguay. Like epitope 595988, RpoA’s T cell epitope 597585 has been validated in a bovine interferon gamma release assay^38^. The substitution is not one believed associated with drug resistance, and while the physiochemical differences between aspartic and glutamic acids are minimal, this change may influence immune recognition, or even still incur minor fitness costs given the fundamental role RpoA plays. Regardless, it seems unlikely this epitope would be a sole major driving factor of observed attenuation in Ravenel and might instead be a contributor among a constellation of changes.

A 4th single amino acid mismatch in Ravenel, in epitope 163423, was found to be in a fragmented, assembled sequence of EsxJ. This epitope mismatch was discarded as unreliable. Subsequently, the last 1aa mismatch (epitope 163642) was found to involve this same gene and was also discarded. Finally, the 3aa mismatch in Ravenel was determined to fall in a partial PPE family protein (WP_152345480.1) that aligns ambiguously with multiple possible PE/PPE genes. Due to the unreliable nature of these genes in sequencing and assembly, this match was disregarded.

Only one unique single epitope change was observed in the virulent strain 10-7428, a modification of PPE42 (Mb2640 in MBO). This protein is known to be highly immunogenic and stimulates a strong humoral response in human patients^43^ and is one of the selected antigens in the ID93 subunit vaccine^44,45^. It is suggested that variation in some PE/PPE genes, unlike in most other antigens, may actually be beneficial to MTB in avoiding a productive, Th1-dominant host immune response^44^. In strain 10-7428, epitope 10022, one of three recognized in PPE42, presented with Asn232Asp. This modification is seen in only one other sequenced isolate. Interestingly, 10022 is a validated B cell epitope unlike the exclusive T cell epitopes changes seen in Ravenel. In contrast with other PE/PPE family genes analyzed in this work, PPE42 is a relatively distinct gene and sequencing, and placement of this substitution is clean in the Ravenel assembly. However, as no in vitro crystal structure has been obtained for this protein and PE/PPE genes are often poorly understood regardless, any interpretation of this finding must remain limited absent further investigation in vitro.

In Ravenel, an additional epitope change was reported that also arose from an atypical assembly pattern around *esxJ*, and this epitope was discarded as an artifact.

The Antigen 85B complex has been known for decades to be a dominant secretion product and immune stimulator^46,47^. Antigen 85B within the MTBC is strongly conserved, with more variation across NTM but noted antibody cross-reactivity between all variants^48^. In BCG-1 Russia, a single substitution was observed, Phe140Leu, that resulted in a change that appears fixed across all BCG strains, with very few non-BCG examples from lab or clinical strains. This single amino acid change modifies four characterized epitopes (149353, 196212, 16926, and 18898) that all overlap this position.

MarP is a serine protease involved in maintenance of intracellular pH during phagocytosis through the processing of peptidoglycan through its interactions with RipA^49^. Loss of MarP or its catalytic residues leave the bacterium severely impaired for pathogenesis in vitro and in a murine model of infection^50,51^. In BCG-1 Russia, epitope 600022 of MarP carried Asn165Thr change, a mutation in the flexible linker region between transmembrane anchor and protease domain. Like the Ag85B mutation, this substitution is also fixed across BCG strains and appears again only in the same small number of lab and clinical isolates, raising the possibility these isolates may be evolutionarily similar to BCG, or cases of BCG-osis.

Mce (mammalian cell entry) family proteins are a class of protein involved with entry into host cells and interference with host cell signaling pathways, particularly through the ERK1 and ERK2 MAPK pathway for inducing cytokine production ^52^. T cell epitope 20707 presented with Thr46Pro. At a molecular level, the sequence change is another significant structural alteration, though whether this affects the function of this protein or its immune recognition in BCG-1 are beyond the scope of this work.

PE/PPE family changes were numerous in BCG-1, but as before, they are difficult to rely on and are reported only with the caveat that their true sequence and placement in the assembly are unclear.

Finally, in silico predictions for ΔΔG were variable and conflicting (Table 2), a known problem in the field^53^. The use of these tools may also be complicated by the specific portions of molecules being studied—surface residue substitutions have been shown previously to be more poorly predicted than changes to residues buried within the protein^54^, and epitopes herein were exposed residues. Despite this, a message remains in these contradictory findings: no consensus changes of a magnitude > 0.5 kcal/mol in either a stabilizing or destabilizing direction was measured for any affected protein in Ravenel or 10-7428, and it is therefore less likely that any observed substitutions are sufficient to modify an affected protein enough to ablate functionality. This investigation is premised on the idea that changes to epitope recognition can lead to dysregulation of host manipulation by *Mycobacterium* species. Indeed, if mutant proteins retain their molecular functionality but still alter virulence, modified epitope recognition is a means of describing this contradiction.

Using epitope data gleaned largely from *M. tuberculosis* var. *tuberculosis*, and after sequencing a uniquely attenuated *M. tuberculosis* var. *bovis* genome in strain Ravenel, the haystack of 4130 epitope alignments yielded 3 needles of epitopes with non-synonymous changes in characterized epitopes. Two of these epitopes have been validated in bovine IFN-γ release assays, and one in a murine model showing IFN-γ and TNF-α release. As recent work has demonstrated, MBO Ravenel provokes potent cell-mediated immune responses in the bovine host, but fails to produce granulomatous lesions in nearly all cases, and the process of an infection leading to granuloma formation is known to be tightly associated with the precise presentation of specific T cell epitopes at specific times^13,20^. In contrast to these, an isolate from a dairy cattle outbreak—MBO 10-7428—was found to contain no T cell epitope variation, and instead the only change fell in a B cell epitope in a gene where variation is associated with increased virulence. These results warrant the further investigations into how these changes in T cell epitopes may be an indicator of attenuation in MBO Ravenel and other strains, an investigation of their role in pathogenesis, and whether any of these epitopes may prove useful as subunits for vaccine development, as has already been proposed for RpfB^34^. An essential next step towards validating the biological implications of this work would be in the testing of wild-type vs. altered epitopes in a bovine T cell assay utilizing samples from previously infected cattle. Detectable differences, positively or negatively, in T cell activation between these epitopes would support a role in attenuation and merit closer scrutiny into the immunological effects. The analytic process described here is simple, and with limited development could be readily incorporated into standard comparative genomics analysis for MTBC organisms, increasing the amount of information researchers can extract from each experiment.

## Materials and methods

### Immune epitope database (IEDB) data accession and filtering

The US National Institutes of Health and the Department of Health and Human Services jointly support and operate the Immune Epitope and Analysis Database (IEDB), a comprehensive database of over 1.5 million experimentally validated epitopes from a range of species and diseases^55^. The IEDB (accessed September 2022) was used to collect all epitopes validated in the *Mycobacterium tuberculosis* complex (ID:77643, n = 4894), primarily from *Mycobacterium tuberculosis* var. *tuberculosis* (n = 4111) and *M. tuberculosis* var. *bovis* (n = 783), including 356 M*. tuberculosis* var. *bovis* BCG strains^55^. These sequences include non-peptide targets like LAM which were removed (n = 46), B cell-specific discontinuous peptide sequences (n = 2), as well as peptides that had undergone post-translational modifications (n = 28), both because they are unable to be processed through tblastn and because the presence or absence of these modifications could definitionally not be verified by screening against whole genome sequences.

### Mapping epitopes to WGS data by TBLASTN

The remaining 4818 sequences were converted to FASTA format and uploaded to the High-Performance Computing Center at Michigan State University (Supplemental File 1: Peptide Sequences). TBLASTN v2.10.0 + was utilized with custom databases built from four genome assemblies^56,57^. Two of these strains of *M. tuberculosis* var. *bovis* were sequenced and analyzed by our lab: attenuated Ravenel (GCF_018305025.1) and virulent 10-7428 (GCF_018305045.1)^19^. From existing databases, the attenuated reference BCG-1 (Russia) (GCF_001483905.1), and virulent reference AF2122/97 (GCF_000195835.3) were selected. The BLAST + package (v2.11.0)^57^, preinstalled on the MSU HPCC, was loaded and the makeblastdb command used with default parameters to generate local databases based on each of the four genomes mentioned. All tblastn searches were with default BLAST parameters, with the filtered list of 4818 epitope sequences searched against each genome database^57^. Some highly repetitive epitopes—particularly those from PE/PPE genes—were caught by filtering, Karlin-Altschul parameters were not calculated by the BLAST algorithm, and they were subsequently excluded from analysis. The tblastn output was produced twice, once in pairwise-alignment format, and once in tabular format. After processing below, epitopes that were unique to our dataset were also TBLASTN searched against the broader NCBI WGS database to determine their presence or absence across all sequenced strains.

### Output filtering and processing

Tables were viewed in Excel, sorted by number of mismatches, sequences with gaps were excluded, and the subsets of homology hits with 0, 1, 2, or 3 mismatches separated into their own respective sheets. The XLOOKUP function was used to find whether any mismatched epitopes had a 100% match against any variant epitope in the Match sheet, and epitopes that appeared only to vary from the IEDB listed sequences—that is, epitopes that truly do not perfectly match known epitope sequences in IEDB—were moved into their own sheets. Finally, for strains Ravenel, 10-7428, and BCG-1, the list of epitopes that varied from IEDB sequences was queried against the same list of TBLASTN searches from MBO reference strain AF2122/97 in order to identify and dismiss candidate epitopes that were identical to the MBO reference and may simply represent the MBO genetic background. The pairwise BLAST results list was then analyzed for each remaining mismatch, epitope IDs queried on IEDB for details, and results recorded. Finally, BLAST searches were also performed with the full-length proteins against their H37Rv and AF2122/97 counterparts to explore potential compensatory mutations. A schematic representation of this workflow is presented in Fig. 1.

### In silico protein stability prediction software

Unique epitopes in Ravenel, 10-7428, and BCG-1 Russia were queried through UniProt and NCBI, and effects on protein stability examined by three high-performing software tools benchmarked by Pancotti et al*.* ^58^(2022)—DDGun3D, PremPS, and INPS—as well as an additional recently released tool, DynaMut2, using in vitro crystal structures preferentially and DeepMind’s AlphaFold in silico predictions when in vitro structures were not available^25–29^.

## Supplementary Information

Supplementary Information.

## Data Availability

Genome data used in this study are available through NCBI BioProject PRJNA713797. GenBank accession numbers are as follows: for raw reads, SRX10318108 for Ravenel and SRX10318109 for 10-7428; for post-PGAP sequences, JAGEUB000000000.1 for Ravenel and JAGEUC000000000.1 for 10-7428. All analyzed data are presented in tables, figures, or supplemental files in this study.
